# Arginine methylation of hnRNPUL1 regulates interaction with NBS1 and recruitment to sites of DNA damage

**DOI:** 10.1038/srep10475

**Published:** 2015-05-28

**Authors:** Gayathri Gurunathan, Zhenbao Yu, Yan Coulombe, Jean-Yves Masson, Stéphane Richard

**Affiliations:** 1 Terry Fox Molecular Oncology Group and Segal Cancer Center, Bloomfield Center for Research on Aging, Lady Davis Institute for Medical Research and Departments of Oncology and Medicine, McGill University, Montréal, Québec, Canada H3T 1E2; 2 Genome Stability Laboratory, Laval University Cancer Research Center, Hôtel-Dieu de Québec (CHUQ), Quebec city, Quebec, Canada, G1R 2J6

## Abstract

Arginine methylation is a post-translational modification required for the maintenance of genomic integrity. Cells deficient in protein arginine methyltransferase 1 (PRMT1) have DNA damage signaling defects, defective checkpoint activation and extensive genomic instability. Herein we identify the DNA damage protein and RNA binding protein, hnRNPUL1, to be a substrate of PRMT1. We identify the dimethylation of R584, R618, R620, R645, and R656, as well as the monomethylation of R661 R685 and R690 within hnRNPUL1 in U2OS cells by mass spectrometry. Moreover, we define the arginines within the RGG/RG motifs as the site of methylation by PRMT1 both *in vitro* and *in vivo*. The arginines 612, 618, 620, 639, 645, 656 and 661 within the human hnRNPUL1 RGG/RG motifs were substituted with lysines to generate hnRNPUL1^RK^. hnRNPUL1^RK^ was hypomethylated and lacked the ability to interact with PRMT1, unlike wild type hnRNPUL1. Co-immunoprecipitation studies showed that hnRNPUL1^RK^ had impaired ability to associate with the DNA damage protein NBS1. Moreover, hnRNPUL1^RK^ was not recruited to sites of DNA damage, unlike wild type hnRNPUL1, in the presence of transcriptional inhibitors. These findings define a role for arginine methylation during the DNA damage response to regulate protein-protein interactions for the recruitment at sites of damage.

Arginine methylation is a common post-translational modification that takes place in eukaryotic cells^1^,^2^,^3^,^4^. The link between arginine methylation and the DNA damage response pathway was initially observed during a proteomic analysis, where the meiotic recombination 11 homolog (MRE11) protein was identified in immunoprecipitations of methyl-specific antibodies^5^,^6^. MRE11 is a DNA damage sensing protein part of trimeric complex termed the MRN complex with NBS1 and RAD50^7^. The key roles of the RGG/RG motif of MRE11 in double strand break (DSB) repair was determined biochemically to alter the processivity of its exonuclease activity^8^,^9^. A knockin strategy where the MRE11 RGG/RG motif was replaced by a KGG/KG motif showed that the mice are hyper-sensitive to ionizing radiation (IR), as predicted for a hypomorphic allele of *MRE11*^10^. It is now known that several DNA repair proteins including 53BP1^11^,^12^, p53^13^, FEN1^14^, BRCA1^15^, RAD9^16^, and DNA polymerase β are regulated by arginine methylation^17^.

There are 3 main forms of methylated arginine identified in eukaryotes: ω-N^G^-monomethylarginine (MMA), ω-N^G^,N^G^-asymmetric dimethylarginine (aDMA), and ω-N^G^,N’^G^-symmetric dimethylarginine (sDMA). Protein arginine methyltransferases (PRMTs) are conserved from yeast to mammals and there are 9 human PRMTs that have been identified that catalyze the transfer of a methyl group from S-adenosylmethionine to a guanidino nitrogen of arginine^2^. Type I PRMTs (PRMT1, 3, 4, 6 and 8) lead to the production of aDMA, while type II PRMTs (PRMT5 and PRMT9) catalyze the formation of sDMA PRMT7 is a type III enzyme known to generate MMAs^18^. The discovery of lysine demethylases demonstrates that protein methylation is reversible^19^, however, an arginine demethylase remains to be identified^3^.

PRMT1 is the main type I mammalian enzyme with a preference for RGG/RG motifs^4^ and embryos from PRMT1 knockout mice die shortly after implantation at E6.5^20^,^21^. The generation of conditional *PRMT1* knockout mouse embryo fibroblasts (MEFs) revealed that the inducible loss of PRMT1 leads to spontaneous DNA damage, checkpoint defects, hypersensitivity to DNA-damaging agents, chromosomal instability, aneuploidy and polyploidy, suggesting that PRMT1 is a key player in the DDR pathway^21^. PRMT1 substrates in the DDR pathway include MRE11, 53BP1 and BRCA1 (for review^6^. An additional type I enzyme, PRMT6 functions to repress gene activation by methylating histones^22^,^23^,^24^. PRMT6 null mice are viable, but the mouse embryo fibroblasts display premature senescence^25^.

Non-coding RNAs, RNA helicases and RNA binding proteins (RBP) have recently been shown to participate in DNA damage signaling. RBPs including hnRNPK^26^, p54nrb/NONO^27^, hnRNPUL1^28^, RBMX and DDX17^29^ have been identified as participants in the DDR pathway. hnRNPUL1 was shown to bind with NBS1 and CtIP^28^, whereas the mechanism of action of RBMX and DDX17 is unknown^29^. The identification of non-coding RNAs at DNA breaks^30^ and its requirement for DNA repair^31^ and 53BP1 recruitment^32^, defines a role for RNA and RBPs during DNA repair. Thus, the role and regulation of RNA, RBPs and ribonucleoprotein complexes at DSBs is unknown.

hnRNPUL1 is known as an hnRNPU-like protein and belongs to the hnRNP family. It was first identified as an adenoviral early region 1B-associated protein 5 (E1B-AP5), since it was known to associate with the adenovirus early protein E1B-55 kDa (Ad5EE1B55K) during lytic infection^33^. hnRNPUL1 binds to the MRN complex and is recruited to the damage site to participate in DSB repair^28^. Specifically, hnRNPUL1 has a RGG/RG motif at its C-terminus that is required to associate with NBS1 and recruit it to DNA damage sites^28^. hnRNPUL1 was shown to function downstream of MRN and CtIP in the DNA resection pathway and induce DNA resection with the recruitment of the BLM helicase^28^. It has been demonstrated that hnRNPUL1 is methylated^34^,^35^, however, the precise methylated arginine residues and the functional implication of the methylation have remained undefined. Herein, we demonstrate that arginine methylation of hnRNPUL1 is required for its association with NBS1 and recruitment to the DNA damage sites.

## Materials and Methods

### Antibodies, immunoprecipitations and immunoblotting

Rabbit anti-hnRNPUL1 antibody was purchased from Proteintech (Chicago, IL). Mouse anti-FLAG (M2) antibody, anti-β-tubulin antibody, and protein-A-Sepharose beads were purchased from Sigma (St. Louis, MO). Anti-GFP antibody was purchased from Novus Biologicals (Littleton, CO). Rabbit anti-PRMT1 antibody and ASYM25b were described previously^21^,^36^. Immunoprecipitations and immunoblotting were performed as previously described^37^. Briefly, cells were lysed in 50 mM HEPES pH 7.4, 150 mM NaCl, and 1% Triton X-100 on ice for 15 min. After removal of the Triton insoluble matter by centrifugation, the supernatant was incubated with the indicated antibodies on ice for 2 h. The bound proteins were immunopurified using protein A Sepharose beads tumbled at 4 °C for 1 h and separated by SDS-PAGE, transferred to nitrocellulose membranes and immunoblotted with the indicated antibodies, as previously described^37^.

### Plasmids

The human hnRNPUL1 cDNA purchased from ORIGENE (Rockville, MD) was FLAG epitope-tagged and subcloned into pcDNA3.1 with the following primers 5′-GGG GGA TCC GAT GTG CGC CGT CTG AAG GTG-3′ and 5′-GGG GTC GAC CTA CTG TGT ACT TGT GCC ACC-3. FLAG-hnRNPUL1^RK^ (R612K, R618K, R620K, R639K, R645K, R656K and R661K) in pcDNA3.1 was generated from FLAG-hnRNPUL1 by Mutagenex Inc (Hillsborough, NJ). GFP-hnRNPUL1 and GFP-hnRNPUL1^RK^ were also generated by Mutagenex Inc. DNA constructs were entirely sequenced. Oligonucleotide sequences used to generate the GST-hnRNPUL1 fragments are as follows:

Di-RG: 5′- GAT CTA TGA AGA AAA CCG GGG ACG GGG GTA CTT TGA GCA CTGA-3′ and 5′-TCG ATC AGT GCT CAA AGT ACC CCC GTC CCC GGT TTT CTT CAT A-3′. RRGR: 5′-GAT CCA CCG AGA GGA TAG GAG GGG CCG CTC TCC TCA GCC TTG A-3′ and 5′-TCG ATC AAG GCT GAG GAG AGC GGC CCC TCC TAT CCT CTC GGT G-3′. RIRG: 5′-GAT CCC CCT TAG TGA GCG TAT CCG GGG CAC CGT TGG ACC ATG A-3′ and 5′-TCG ATC ATG GTC CAA CGG TGC CCC GGA TAC GCT CAC TAA GGG G-3′.

Tri-RG: 5′-GAT CTT TGA CAA CCG AGG TGG TGG TGG CTT CCG GGG CCG CGG GGG TGG TGG TGG CTT CCA GTG A-3′ and 5′- TCG ATC ACT GGA AGC CAC CAC CAC CCC CGC GGC CCC GGA AGC CAC CAC CAC CTC GGT TGT CAA A-3′.

Di-RGG: 5′-GAT CCC TGG AGG CAA CCG TGG CGG CTT CCA GAA CCG AGG GGG AGG CAG CGG TGG AGG ATG A-3′ and 5′-TCG ATC ATC CTC CAC CGC TGC CTC CCC CTC GGT TCT GGA AGC CGC CAC GGT TGC CTC CAG G-3′.

Mono-RGG: 5′-GAT CGG AGG AGG CAA CTA CCG AGG AGG TTT CAA CCG CAG CGG AGG TGG TGG CTG A-3′ and 5′-TCG ATC AGC CAC CAC CTC CGC TGC GGT TGA AAC CTC CTC GGT AGT TGC CTC CTC C-3′. All glutathione S-transferase (GST) proteins were isolated, as described previously^36^.

### Cell culture, transfection and drug treatments

HEK293 and U2OS cell lines were purchased from the American Type Culture Collection (Manassas, VA). Cells were cultured in Dulbecco’s modified Eagle medium (DMEM; Invitrogen) supplemented with 10% fetal bovine serum (FBS; BioSera), 1 mM sodium pyruvate and antibiotics under typical culture conditions. Plasmids were transfected with Lipofectamine 2000 (Invitrogen), and siRNAs were transfected with Lipofectamine RNAiMAX (Invitrogen) as per the manufacturer’s instructions. siRNAs purchased from Dharmacon Inc. (Lafayette, CO) along with their respective target sequences were as follows: siLuciferase (siCTL, 5′-CGU ACG CGG AAU ACU UCG A dTdT- 3′) and sihnRNPUL1 (5′-GCA GUG GAA CCA GUA CUA U dTdT-3′). siPRMT1 (5′-CGT CAA AGC CAA CAA GTT A dTdT- 3′) was as reported previously to be specific for PRMT1^21^. Cells were analyzed 72 h after transfection.

### *In vitro* methylation assays

The methylation of the hnRNPUL1 RGG/RG motifs by PRMT1 was performed using recombinant proteins, as described^36^. The indicated recombinant GST-hnRNPUL1 protein was incubated with GST-PRMT1 and 0.55 μCi of methyl-^3^H-S-adenosyl-L-methionine in the presence of 25 mM Tris- HCl at pH 7.4 for 1 h in a final total volume of 30 μl. The reactions were stopped by adding 30 μl of 2X Laemmli protein loading buffer, followed by boiling the samples at 100 °C for 10 min. The methylation of the GST-RGG/RG motifs was assessed by polyacrylamide gel electrophoresis and stained with Coomassie Blue. The destained gel was soaked in EN^3^HANCE for 1 h, followed by a gentle agitation in cold water for 30 min. The washed gel was dried at 80 °C for 1.5 h and visualized by fluorography.

### Laser irradiation and confocal microscopy

Immunofluorescence localization of specific proteins at laser-induced DSBs *in vivo* was performed as described previously^38^.

## Results

### Identification of methylated arginine residues of hnRNPUL1 in U2OS cells

To identify the methylated arginines within hnRNPUL1, an expression vector encoding FLAG-epitope tagged hnRNPUL1 was transfected in U2OS cells. The cells were lysed under denaturing conditions to identify the post-translational modifications of hnRNPUL1, and not its co-immunoprecipitating proteins. Seventy peptides with 62% coverage of hnRNPUL1 and 42 of the total 66 arginines were detected by mass spectrometry (Supplementary Table1 and Supplementary Figure 1). hnRNPUL1 C-terminus residues 732 to 856 do not contain lysines or arginines and, therefore, were not detected in our analysis. We identified arginines 661, 685 and 690 to harbor monomethyls, while arginines 584, 618, 620, 645 and 656 were dimethylated (Table 1). Arginines 618, 620, 645 and 656 are located within the RGG/RG motifs^4^. Moreover, four out of the five monomethylated arginines contain an N-terminal asparagine (Table 1).

### PRMT1 methylates and associates with hnRNPUL1 *in vivo*

**Figure 1:**
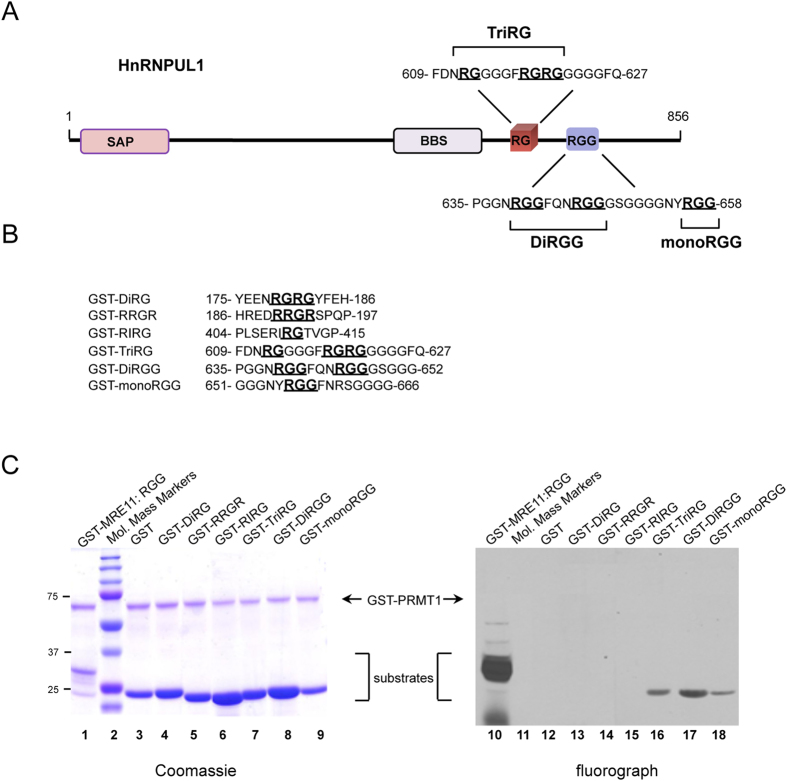
The RGG/RG motif of hnRNPUL1 is methylated by PRMT1 *in vitro*. **A)** SAP designates the SAF- A/B, Acinus and PIAS motif, while BBS denotes BRD7-binding site. The RGG/RG motifs located from 609 to 658 are shown. The RG and RGG repeats are bold and underlined. **B)** The human hnRNPUL1 peptide sequences fused to recombinant GST used for the methylation assay of *panel C*. **C)** PRMT1 *in vitro* methylation assay using GST-hnRNPUL1 proteins of panel B with H^3^-SAM. Proteins were resolved by SDS-PAGE, stained with Coomassie blue (left panel), dried and visualized by fluorography (right panel). GST-MRE11:RGG (glycine arginine rich) and GST were used as the positive and negative control, respectively. The indicated sequences (RGG or RG) fused to GST migrate at a similar molecular mass as GST alone because of the short added sequence. GST-MRE11:RGG harbors an extra ~60aa and migrates close to 33 kDa. The migration of GST-PRMT1 is shown with arrows.

PRMT1 is the major arginine methyltransferase in mammalian cells and it has a preference is for arginines in RG/RGG motifs^4^. We examined *in vitro* whether the hnRNPUL1 RGG/RG motifs were indeed substrates of PRMT1. hnRNPUL1 peptide sequences inserted in-frame with glutathione S-transferase (GST, Fig. 1A,B) were incubated with GST-PRMT1 and *methyl*-^3^H-S-adenosyl-L-methionine for an *in vitro* methylation assay. The proteins were stained with Coomassie Blue to confirm their equivalent expression (Fig. 1C, lanes 1 and 3 to 9) and the methylated proteins were visualized by fluorography (Fig. 1C, lanes 10 and 12 to 18). The GST-TriRG, GST-DiRGG, and GST-monoRGG proteins were methylated by PRMT1 (Fig. 1C, lanes 16-18). In contrast, the GST-DiRG, GST-RRGR, and GST-RIRG proteins were not methylated (Fig. 1C, lanes 13-15). The RGG/RG motif of MRE11 was used as a positive control and GST alone, as a negative control, respectively (Fig. 1C, lanes 10 and 12).

**Figure 2:**
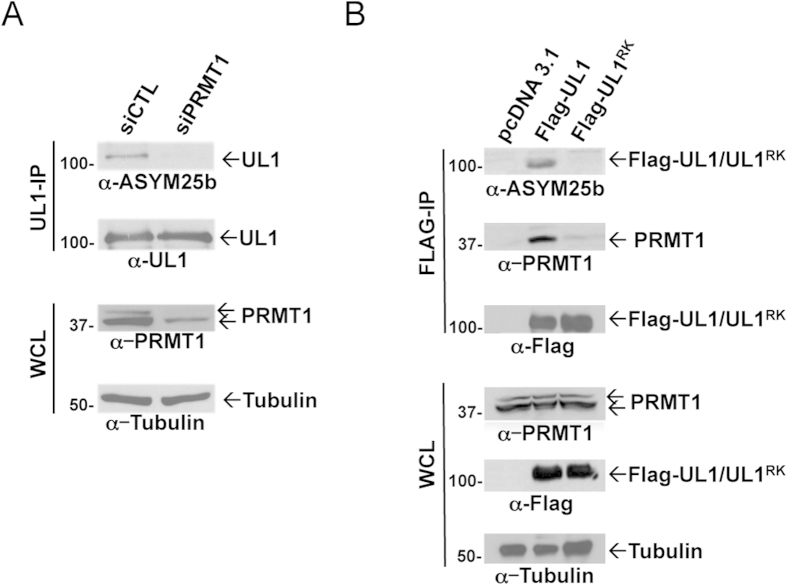
The RGG/RG motif of hnRNPUL1 is methylated by PRMT1 *in vivo*via a physical association. **A)** Whole cell lysates from HEK293 cells transfected with siControl (CTL) and siPRMT1 were subjected to immunoprecipitation (IP) 48 h post-transfection with the anti-hnRNPUL1 antibody. Whole cell lysates (WCL) and immunoprecipitants were immunoblotted with anti-asymmetrical dimethylarginines (ASYM25b), anti-hnRNPUL1, anti-PRMT1 and anti-Tubulin antibodies. **B)** HEK293 cells transfected with wild type FLAG-hnRNPUL1 and FLAG-hnRNPUL1^RK^ were lysed and anti-FLAG immunoprecipitations were performed. The bound proteins were separated by SDS-PAGE and immunoblotted with the indicated antibodies. Tubulin was used as loading control.

We next examined whether PRMT1 was the enzyme responsible for the methylation of endogenous hnRNPUL1. HEK293 cells were transfected with siRNAs targeting luciferase (siCTL) or PRMT1. Cellular extracts were prepared for immunoprecipitation with anti-hnRNPUL1 antibodies and the bound proteins were separated by SDS-PAGE and immunoblotted with ASYM25b, a specific asymmetric dimethylarginine antibody^10^. hnRNPUL1 was recognized by anti-ASYM25b antibodies in siCTL, but not siPRMT1-transfected cells, suggesting that hnRNPUL1 is hypomethylated in PRMT1-depleted cells (Fig. 2A, α-ASYM25b panel). Equivalent hnRNPUL1 was immunoprecipitated from siCTL and siPRMT1 cells (Fig. 2A, α-UL1 panel). Anti-PRMT1 immunoblotting of whole cell lysates (WCL) confirmed the PRMT1 knockdown and anti-tubulin immunoblotting confirmed equivalent loading (Fig. 2A, lower panels). These findings suggest that PRMT1 is the physiological enzyme responsible for the arginine methylation of endogenous hnRNPUL1.

We next proceeded to substitute all seven arginines located within the two RGG/RG motifs that are likely methylated by PRMT1 (R612K, R618K, R620K, R639K, R645K, R656K and R661K; hnRNPUL1^RK^; Fig. 1A). We initially examined whether FLAG-hnRNPUL1^RK^ was hypomethylated *in vivo*. HEK293 cells were transfected with either FLAG-hnRNPUL1 or FLAG-hnRNPUL1^RK^. The cells were lysed, immunoprecipitated with anti-FLAG antibodies and the presence of methylation was visualized by immunoblotting with ASYM25b. hnRNPUL1, but not FLAG-hnRNPUL1^RK^, was arginine methylated (Fig. 2B,α-ASYM25b panel). These findings indicate that the arginines that reside within the RGG/RG motifs are the main methylated residues of hnRNPUL1 recognized by ASYM25b antibodies.

We then tested whether endogenous PRMT1 associated with FLAG-hnRNPUL1 and FLAG-hnRNPUL1^RK^. HEK293 cells transfected with FLAG-hnRNPUL1 or FLAG-hnRNPUL1^RK^ were lysed and co-immunoprecipitation analyses were performed. We observed that PRMT1 co-immunoprecipitated with FLAG-hnRNPUL1, but not FLAG-hnRNPUL1^RK^ (Fig. 2B, α-PRMT1 upper panel). The fact that PRMT1 did not associate with FLAG-hnRNPUL1^RK^ (Fig. 2B, α-PRMT1 upper panel), likely explains its hypomethylation, as PRMT1 is known to associate with its substrates^39^. These findings suggest that the arginines located within the RGG/RG motifs of hnRNPUL1 are sites of PRMT1 methylation.

### Arginine methylation regulates the interaction of hnRNPUL1 with NBS1

**Figure 3:**
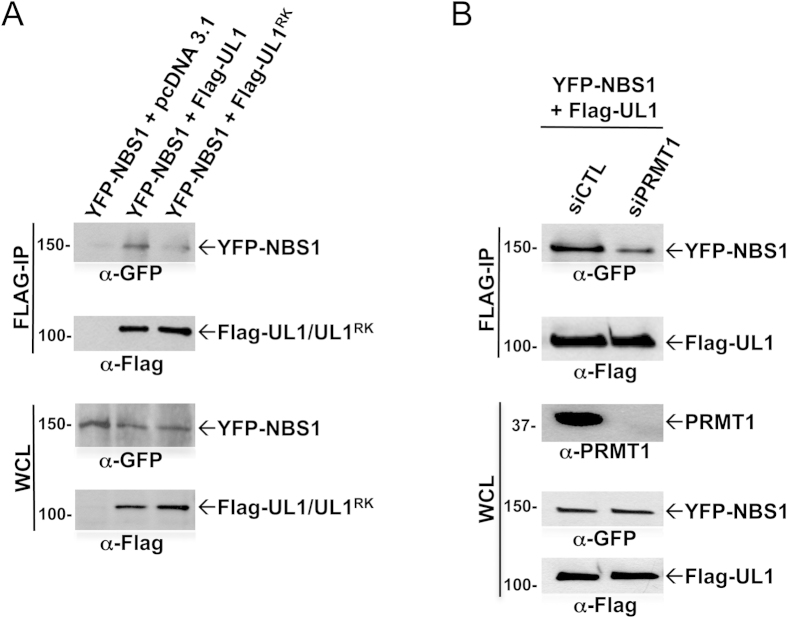
Methylation of hnRNPUL1 is required for its interaction with NBS1. **A)** U2OS cells were transfected with empty vector (pcDNA 3.1), FLAG-hnRNPUL1 wild type, FLAG-hnRNPUL1^RK^ mutant and YFP-NBS1. Whole cell lysates (WCL) and FLAG-immunoprecipitants were immunoblotted with the indicated antibodies. **B)** HEK293 cells were transfected with siControl and siPRMT1 along with YFP-NBS1 and FLAG-hnRNPUL1. The whole cell lysates (WCL) and FLAG immunoprecipitates were subjected to western blot analysis using the antibodies indicated.

hnRNPUL1 interacts with NBS1, a component of the DNA damage sensing complex MRE11-RAD50-NBS1^28^. To determine whether arginine methylation of hnRNPUL1 affects its interaction with NBS1, U2OS cells were co-transfected with a control pcDNA3.1, FLAG-hnRNPUL1 or FLAG-hnRNPUL1^RK^ and YFP-NBS1 expression vectors. The cells were lysed, anti-FLAG antibody immunoprecipitations were performed and the bound proteins were immunoblotted with anti-GFP antibodies. YFP-NBS1 associated with the FLAG-hnRNPUL1, but had a reduced affinity for FLAG-hnRNPUL1^RK^ (Fig. 3A, upper panel). To further determine whether arginine methylation of hnRNPUL1 regulates its interaction with NBS1, we performed co-immunoprecipitation assays in PRMT1-deficient cells. HEK293 cells were transfected with siRNAs for luciferase (siCTL) or PRMT1 along with expression vectors for YFP-NBS1 and FLAG-hnRNPUL1. The cells were immunoprecipitated with anti-FLAG antibodies and the bound proteins were separated by SDS-PAGE and immunoblotted with anti-GFP antibodies. We observed a reduced interaction between NBS1 and hnRNPUL1 in siPRMT1 transfected cells compared to siCTL cells (Fig. 3B). These findings suggest that arginine methylation by PRMT1 regulates the interaction between hnRNPUL1 and NBS1.

### The methylation of the hnRNPUL1 RGG/RG motifs regulates its recruitment at sites of DNA damage

**Figure 4:**
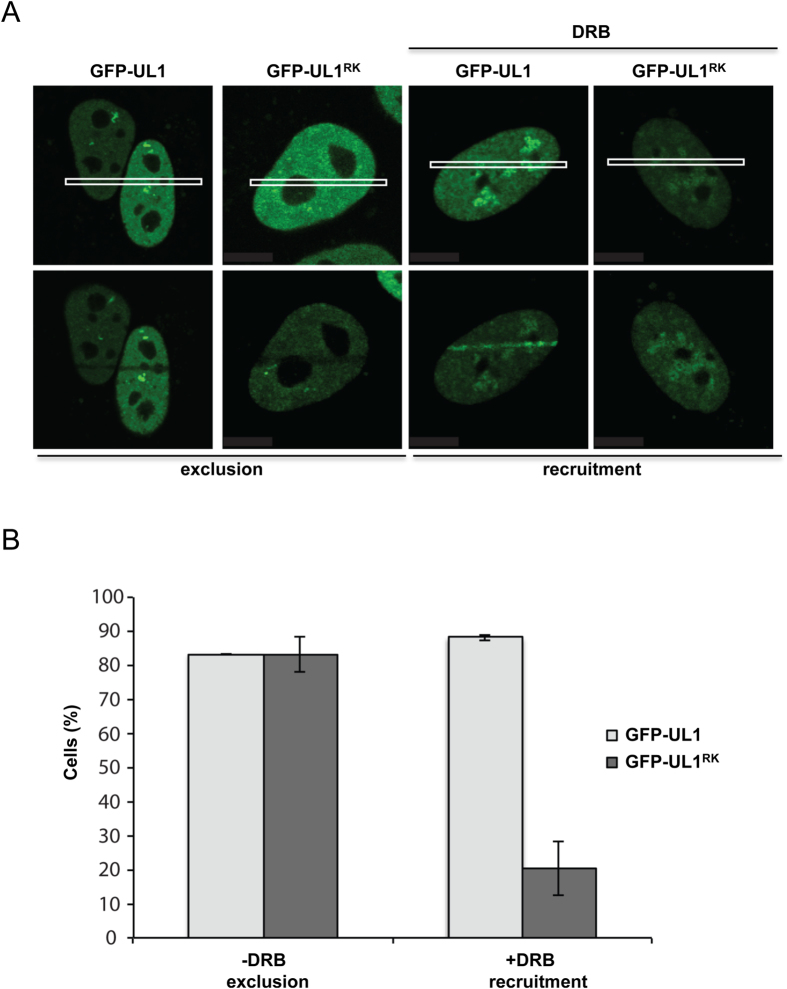
RGG/RG motif regulates GFP-hnRNPUL1 recruitment at breaks. **A)** U2OS cells were transfected with wild type GFP-hnRNPUL1 and GFP-hnRNPUL1^RK^ and subjected to laser micro-irradiation treatment (forms a DNA damage ‘track’; white box). Exclusion of GFP-UL1 proteins from laser damage sites (2 min post-laser scissor damage) was monitored via live imaging (exclusion) or recruitment in the presence of DRB (inclusion) **B)** Quantification of exclusion and inclusion patterns of GFP-UL1 proteins are indicated as a percentage.

hnRNPUL1 exhibits two types of dynamics at DNA damage sites depending on the presence or absence of RNA^28^. hnRNPUL1 is rapidly excluded from laser microirradiation sites, but in the presence of transcription inhibitors, it is effectively recruited at DNA damage tracks^28^. To assess the role that the RGG/RG motif in recruitment following DNA damage, we transfected U2OS cells with GFP-hnRNPUL1 or GFP- hnRNPUL1^RK^. The cells were examined for recruitment to laser microirradiated nuclear regions in the absence and presence of the transcriptional inhibitor, 5,6 dichloro-β-D-ribofuranosylbenzimidazole (DRB). Both GFP-hnRNPUL1 and hnRNPUL1^RK^ were excluded from laser microirradiated nuclear regions in U2OS cells without DRB (Fig. 4). GFP-hnRNPUL1 was effectively recruited at sites of DNA damage, while GFP-hnRNPUL1^RK^ was not recruited at these DNA damage sites with DRB treatment (Fig. 4). These findings suggest that arginine methylation of the RGG/RG motifs is required for the recruitment at sites of DNA damage in the presence of transcription inhibitors, but are not required for the exclusion of hnRNPUL1 from sites of DNA damage.

## Discussion

In the present manuscript, we identify arginines that are mono- and dimethylated within human hnRNPUL1 in U2OS cells. These methylated arginine residues reside mainly within two separate RGG/RG motifs. *In vivo* and *in vitro* experiments confirmed that PRMT1 was the enzyme responsible for the methylation of these RGG/RG sequences. Replacement of these methylated arginines or deficiency of PRMT1 expression significantly inhibited the association of hnRNPUL1 with NBS1. Furthermore, the ability of FLAG-hnRNPUL1^RK^ to localize to sites of DNA damage was impaired. These findings suggest that methylation of the RGG/RG motifs regulates key protein-protein interactions for chromatin recruitment of hnRNPUL1.

Previous studies have shown that upon laser scissor damage, hnRNPUL1 forms unique exclusion and recruitment patterns^28^. hnRNPUL1 is essentially excluded from sites of DNA damage, however, in the presence of transcription inhibitors, hnRNPUL1 is recruited to them^28^. Our studies revealed that arginine to lysine substitution in the RG/RGG motifs affected hnRNPUL1 recruitment, but not its exclusion (Fig. 4). We demonstrated that arginine methylation of the RGG/RG motifs plays an important role in regulating the association of hnRNPUL1 with the MRN complex, as hypomethylation of hnRNPUL1 had reduced association with NBS1. This may reflect the requirement of the MRN complex for the recruitment of hnRNPUL1 to DSB damage sites. Moreover, it has been reported that hnRNPUL1 lies between MRN and CtIP and BLM, such that hnRNPUL1 promotes DSB resection by regulating BLM recruitment^28^. FLAG-hnRNPUL1^RK^ associated normally with BLM and PRMT1 deficiency did not affect the hnRNPUL1/BLM association (data not shown). This suggests that methylation of hnRNPUL1′s RGG/RG motifs is functionally involved in its association with upstream components, but not necessarily with the downstream effectors in the DDR pathway. It is also possible that NBS1-hnRNPUL1-PRMT1 forms a complex and loss of PRMT1 destabilizes this interaction, without involvement of arginine methylation.

hnRNP proteins have been implicated in numerous cellular processes including RNA metabolism, telomere elongation, DNA repair, and chromatin reorganization^40^. hnRNPUL1 has sequence homology to hnRNPU, also known as scaffold attachment factor A (SAF-A), that is a nucleic acid binding ribonucleoprotein which has a characteristic binding preference for scaffold associated DNA regions including areas rich in A/T stretches^41^,^42^. hnRNPU contains an RGG/RG motif from residue 778 to 793 which is the preferred site for arginine methylation by PRMT1^43^.

Defects in PRMT1-deficient cells have been previously characterized^21^. It has been shown that PRMT1 is essential for early development using a conditional null allele of *PRMT1* in mice, as null embryos die at embryonic day 6.5^20^,^21^. PRMT1-deficient MEFs display genomic instability and exhibit spontaneous DNA damage, checkpoint defects, and delays in cell cycle progression^21^. The arginine methylation of the DNA damage response and RNA binding protein hnRNPUL1 further supports a role for PRMT1 in the DDR pathway.

The role of arginine methylation and PRMTs in the DDR pathway has not yet been fully characterized. Nevertheless, we observed the methylation status of hnRNPUL1 in PRMT1 knockdown cells using siRNA. hnRNPUL1 was shown to be hypomethylated in the PRMT1-deficient cells, demonstrating that PRMT1 is the physiological enzyme responsible for the methylation of RGG/RG motif of hnRNPUL1. We did not observe an increase in the arginine methylation of hnRNPUL1 with DNA damage, suggesting that the regulation is likely with associated proteins (data not shown). Previous studies have implicated hnRNPUL1 to be required for resection at DSBs and to be required for optimal ATR activation. It was proposed that there are two pools of hnRNPUL1 in the cell, such that one pool is involved in RNA metabolism, while the other participates in DSB repair^28^ and that perhaps arginine methylation regulates accessibility between these two pools.

## Additional Information

**How to cite this article**: Gurunathan, G. *et al.* Arginine methylation of hnRNPUL1 regulates interaction with NBS1 and recruitment to sites of DNA damage. *Sci. Rep.* **5**, 10475; doi: 10.1038/srep10475 (2015).

## Supplementary Material

Supplementary Information

## Figures and Tables

**Table 1:** Methylated arginine residues of hnRNPUL1 identified *in vivo* by mass spectrometry.

**Peptide Region**	**Di-methyl (R)**	**Sequence**
613-628	R618, R620	GGGGF**R**G**R**GGGGGFQR
640-656	R645, R656	GGFQN**R**GGGSGGGGNY**R**
563-584	R584	ANFTLPDVGDFLDEVLFIELQ**R**EEADK
Peptide Region	Mono-methyl (R)	Sequence
672-690	R685, R690	WGNNNRDNNNSNN**R**GSYN**R**
640-661	R645, R656, R661	GGFQN**R**GGGSGGGGNY**R**GGFN**R**
