# Supplementary material for: Arginine methylation of hnRNPUL1 regulates interaction with NBS1 and recruitment to sites of DNA damage
Source: Sci Rep. 2015 May 28;5:10475. doi: 10.1038/srep10475 (PMC4447065; doi:10.1038/srep10475)

Arginine methylation of hnRNPUL1 regulates interaction with NBS1 and recruitment to sites of DNA damage

G. Gurunathan, Z. Yu, Y. Coulombe, J. Y. Masson, and S. Richard

Figure 2A

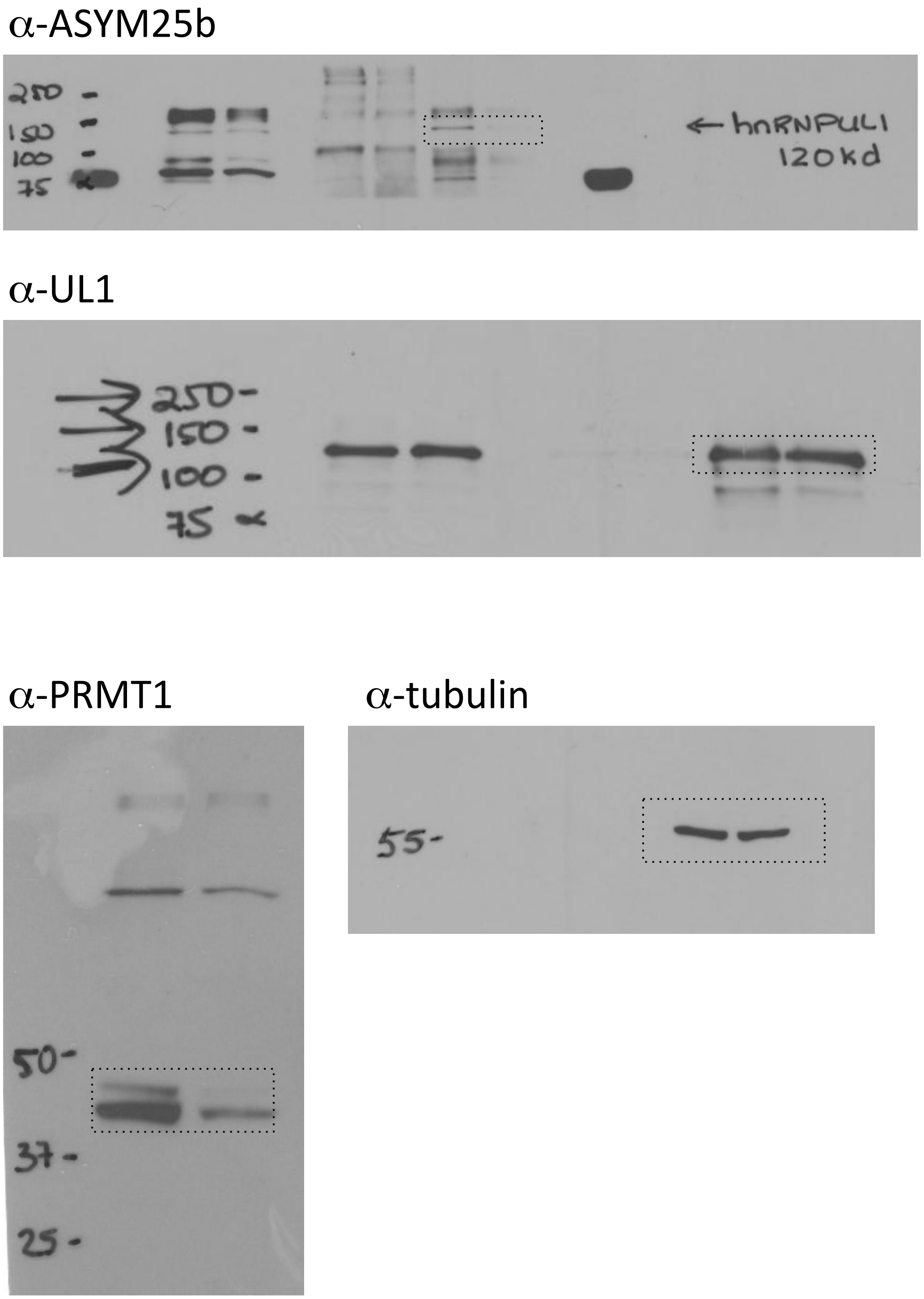

Figure 2A

Arginine methylation of hnRNPUL1 regulates interaction with NBS1 and recruitment to sites of DNA damage

G. Gurunathan, Z. Yu, Y. Coulombe, J. Y. Masson, and S. Richard

Figure 2B

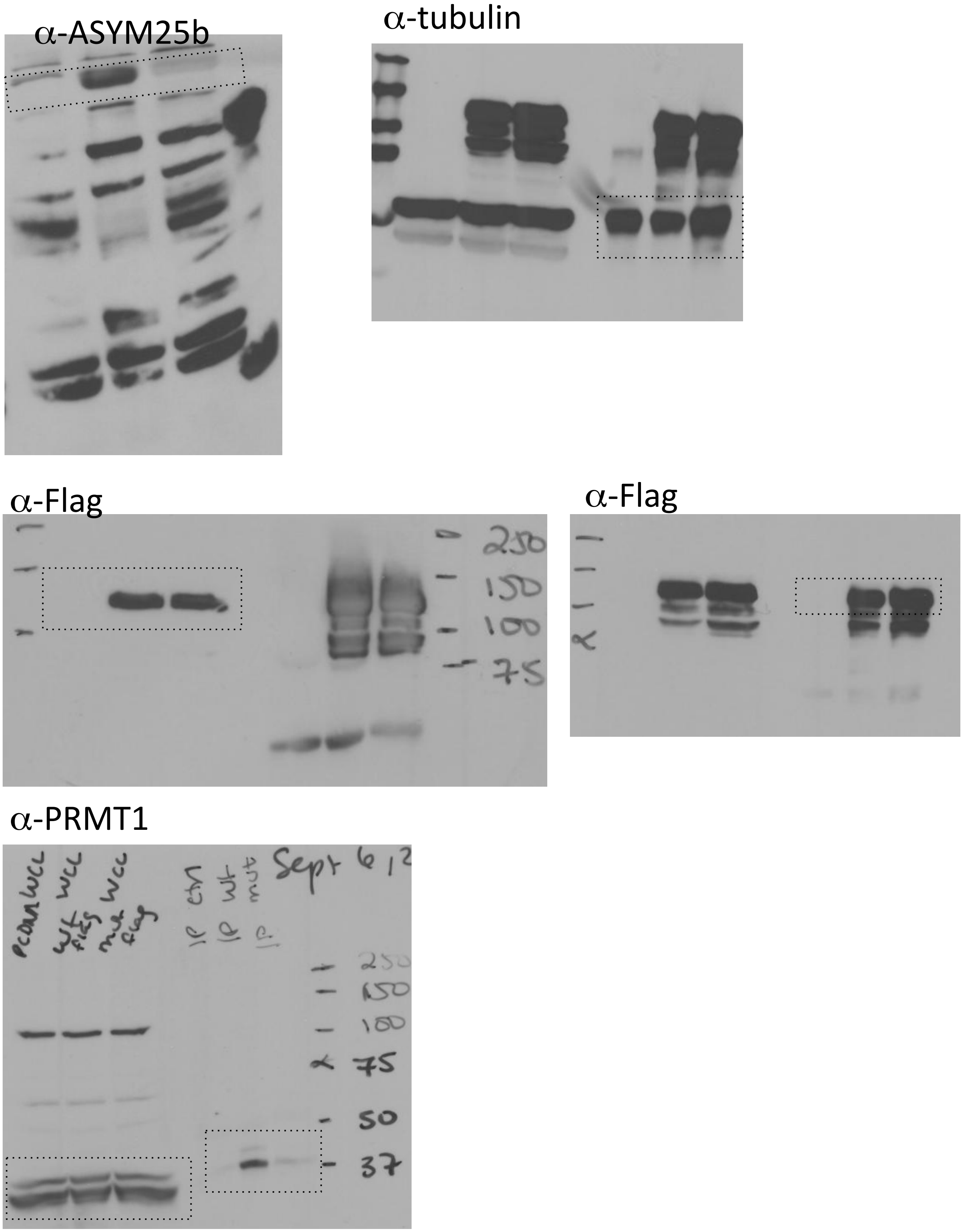

Arginine methylation of hnRNPUL1 regulates interaction with NBS1 and recruitment to sites of DNA damage

G. Gurunathan, Z. Yu, Y. Coulombe, J. Y. Masson, and S. Richard

Figure 3A

α-GFP

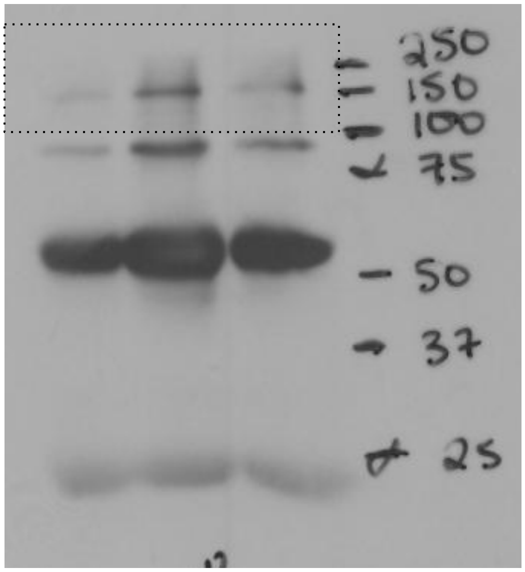

α-FLAG

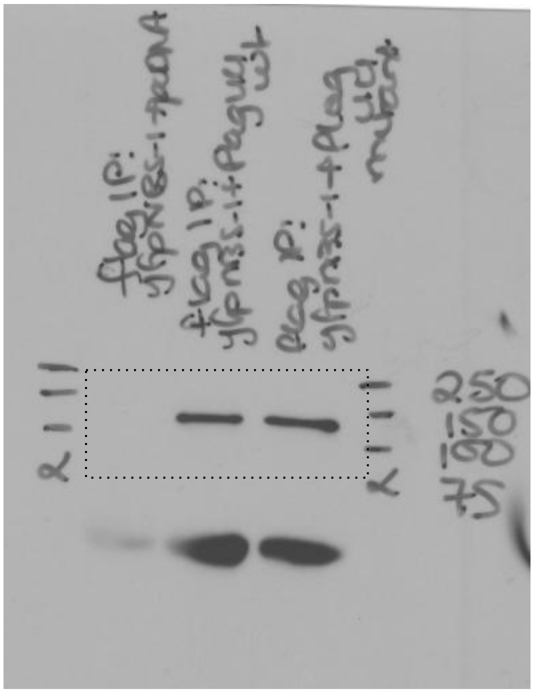

α-GFP

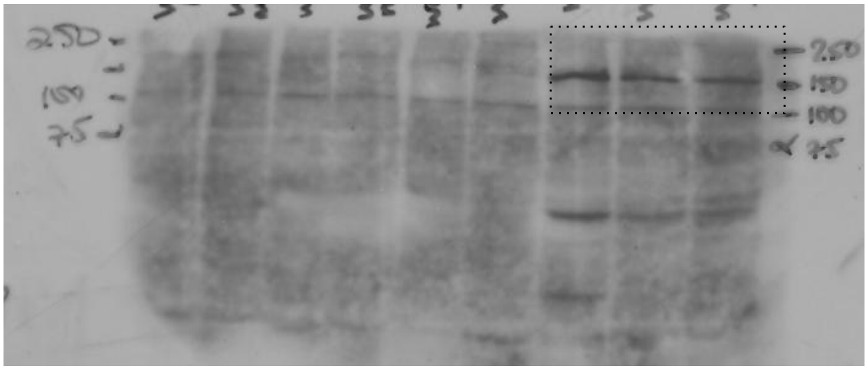

α-FLAG

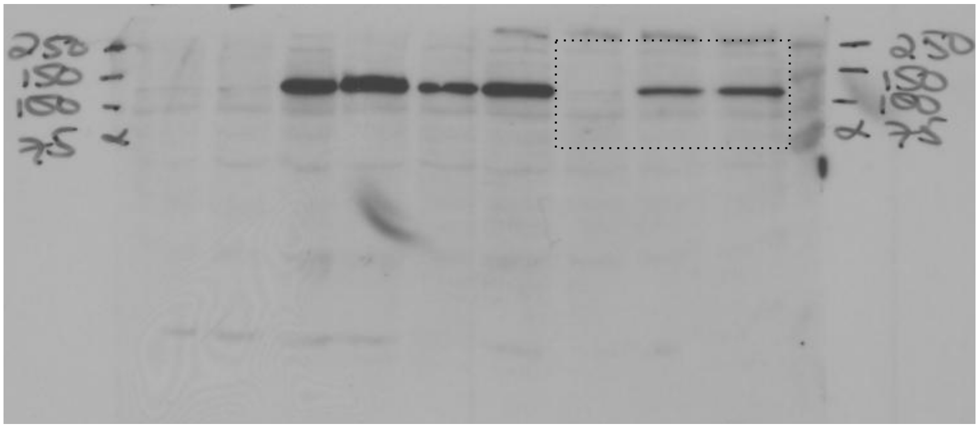

Arginine methylation of hnRNPUL1 regulates interaction with NBS1 and recruitment to sites of DNA damage

G. Gurunathan, Z. Yu, Y. Coulombe, J. Y. Masson, and S. Richard

Figure 3B

α-GFP

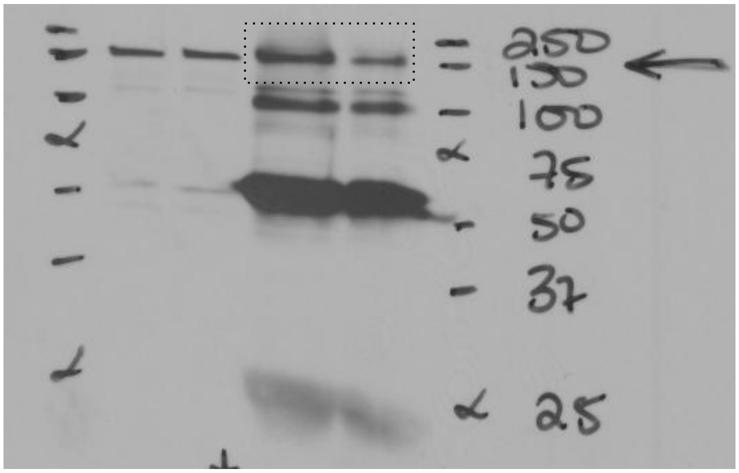

α-Flag

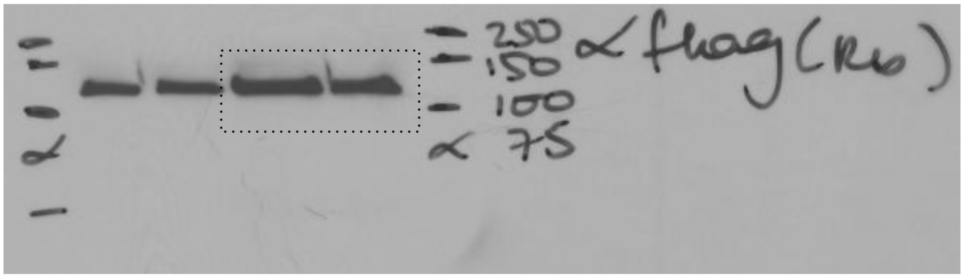

α-PRMT1

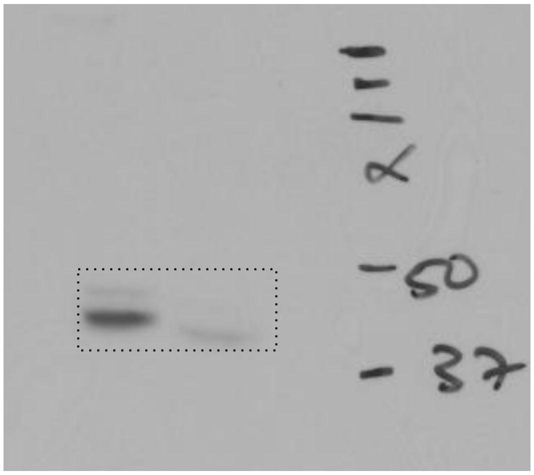

Arginine methylation of hnRNPUL1 regulates interaction with NBS1 and recruitment to sites of DNA damage

G. Gurunathan, Z. Yu, Y. Coulombe, J. Y. Masson, and S. Richard

Figure 3B

α-GFP

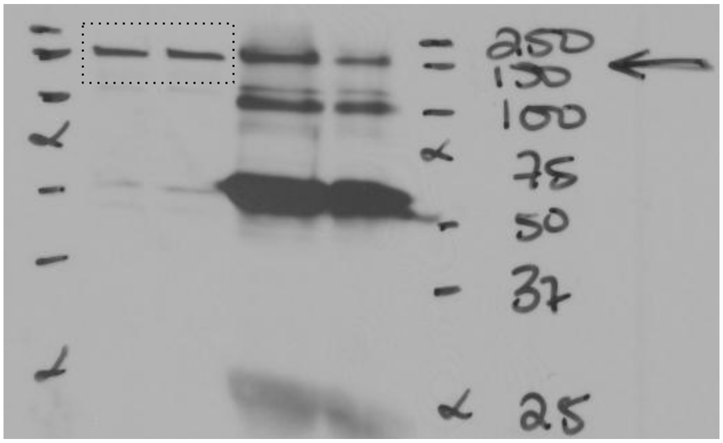

α-Flag

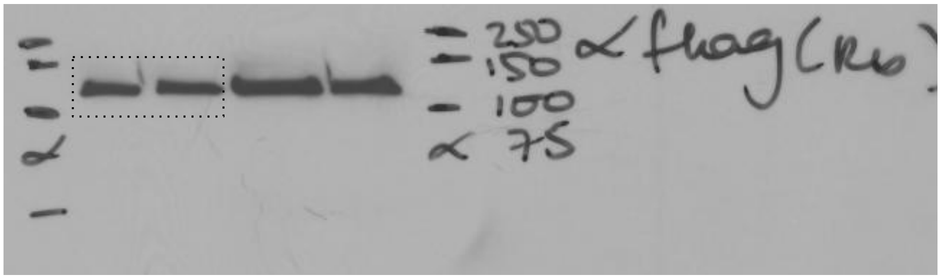

Supplement: Supplementary Information [file srep10475-s1.pdf]
